# Retrospektive Studie zur Auswertung von Blutkulturen bei Pferden im Zeitraum von 2022 bis 2024

**DOI:** 10.1055/a-2848-1852

**Published:** 2026-05-07

**Authors:** Marie-Louise Geisler, Anton Heusinger, Elisabeth Müller

**Affiliations:** 1 Laboklin GmbH und Co. KG, Bad Kissingen

**Keywords:** Bakteriologische Kultur, Bakteriämie, Sepsis, SIRS-Kriterien, Fragebogen, Bacteriological culture, bacteremia, sepsis, SIRS-criteria, questionnaire

## Abstract

**Gegenstand und Ziel:**

In der vorliegenden Studie wurden die Ergebnisse von Blutkulturen von Pferden ausgewertet, welche als Routineproben in den Jahren 2022–2024 von tierärztlichen Praxen an ein veterinärmedizinisches Diagnostiklabor in Deutschland übermittelt wurden. Der Fokus lag auf der Differenzierung der bakteriellen Isolate und wurde durch einen Fragebogen an die Praxen komplementiert, um sepsisassoziierte Symptome zu erfassen und die Ergebnisse in klinischen Kontext einordnen zu können.

**Material und Methoden:**

Die Auswertung der bakteriologischen Kulturen erfolgte retrospektiv anhand der vorliegenden Befundergebnisse. Die insgesamt 199 inkludierten Proben stammten aus Deutschland sowie dem europäischen Ausland. Zusätzlich wurde ein Fragebogen, der unter anderem den Grund für die Durchführung der Blutkultur sowie mögliche sepsisassoziierte Symptome abfragte, an die zugehörigen Praxen versendet und ausgewertet.

**Ergebnisse:**

Der Anteil positiver Blutkulturen lag bei 50,3% (n=100), die sich in 71% monomikrobielle und 29% polymikrobielle Proben aufteilten. Am häufigsten nachgewiesen wurden Staphylokokken (30,2%) gefolgt von Bakterien der Gattung Enterobacterales (18,9%). Knapp die Hälfte der Tiere (41,3%) war zum Zeitpunkt der Probennahme antibiotisch vorbehandelt. Atemwegsinfektionen waren mit 17,4% der am häufigsten genannte Grund für die Einleitung einer Blutkultur.

**Schlussfolgerung:**

Die Ergebnisse der vorliegenden Studie sind mit vorangegangenen Auswertungen vergleichbar. Auf eine Einteilung der Isolate in klinisch relevante Erreger und Kontaminanten wurde verzichtet, da bei dem Großteil der Patienten nur ein Entnahmezeitpunkt vorlag und daher potenziell jedes Isolat als verursachendes Agens infrage kam.

**Klinische Relevanz:**

Sepsis ist kein spezifisches Krankheitsbild und gründet sich auf die klinische Diagnose und das Ergebnis der Blutkultur. Vor dem Hintergrund der Dauer einer bakteriologischen Kultur und des oftmals kritischen Zustands der Patienten ist das Wissen um wahrscheinlich zugrundeliegenden Erreger essenziell, um eine adäquate Therapie schnellstmöglich einleiten zu können.

## Abstract (en)

**Objective:**

In this study, the results of equine blood cultures sampled during the period from 2022 to 2024 were evaluated. Samples were submitted as routine samples by veterinary practices to a veterinary diagnostic laboratory in Germany. The study focused mainly on the differentiation of the bacterial isolates. In order to place the results in a clinical context, the study was complemented by a questionnaire sent to the practices enquiring for sepsis-associated clinical signs.

**Material and methods:**

The study was carried out retrospectively. A total of 199 blood culture samples submitted by practices located in Germany and other European countries were included into the evaluation. A questionnaire asking for the reason of the individual blood collection and possible presence of sepsis-associated clinical signs was sent to the relevant practices and the returning responses were analyzed.

**Results:**

50.3% (n=100) of the blood culture samples showed a positive result, divided into 71% monomicrobial and 29% polymicrobial samples. Staphylococci were most frequently detected (30.2%), followed by bacteria of the genus Enterobacterales (18.9%). Nearly half of the horses (41.3%) was pretreated with antibiotics at the time of sample collection. Respiratory infection was the most frequently listed reason (17.4%) for initiation of a blood culture analysis.

**Conclusion:**

The results of the present study are comparable to previous studies. The isolates were not classified into clinically relevant pathogens and contaminants, as for the majority of patients only one sampling timepoint was available, making any isolate a potential causative agent for bacteremia.

**Clinical relevance:**

Sepsis is not a specific condition, but is based on the clinical findings and the result of the performed blood culture analysis. Considering the duration of a bacteriological culture procedure as well as the commonly critical condition of the patient, availability of data concerning the most frequently occurring pathogens is essential in rapidly initiating adequate treatment.

## Einleitung

Eine Bakteriämie wird definiert als das Vorkommen lebender Mikroorganismen in der Blutzirkulation. Sie tritt bei verschiedenen Infektionsarten auf und ist nicht zwingend mit einer systemischen Entzündungsreaktion verbunden. Bei immunkompetenten Patienten werden zirkulierende Erreger normalerweise durch Zellen des mononukleären Phagozytosesystems (MPS) entfernt, um eine Ausbreitung auf andere Organsysteme zu verhindern. Zudem erkennen Makrophagen und andere Zellen des angeborenen Immunsystems sog. Pathogen-assoziierte molekulare Muster ( *Pathogen-associated molecular patterns* , PAMPs) auf Bakterien, Viren oder Pilzen und initiieren eine zunächst lokale proinflammatorische Reaktion 1 . Klinisch kann sich eine darauf folgende systemische Entzündungsreaktion ( *systemic inflammatory response syndrome* , SIRS) in sogenannten positiven SIRS-Kriterien manifestieren. Dazu gehören u.a. Fieber oder Hypothermie, Tachykardie, Tachypnoe, Leukozytose oder Leukopenie mit oder ohne Linksverschiebung 2 . Während SIRS auch nicht-infektiöse Ursachen haben kann, beschreibt der Begriff Sepsis die fehlregulierte Immunreaktion infolge eines infektiösen Agens, die zur lebensbedrohlichen Organdysfunktion und sogar zum Tod führen kann 1 . In der Humanmedizin werden die Kriterien zur Einteilung des Schweregrads kontinuierlich angepasst. Demnach gilt dort aktuell das als Sepsis-3 bezeichnete Konzept, nach dem der Begriff einer schweren Sepsis eliminiert und der septische Schock als Unterkategorie der Sepsis eingeordnet wird. Die Schwere der Organdysfunktion wird anhand eines als *Sequential Organ Failure Assessment Scores* (SOFA-Score) bezeichneten Punktesystems bewertet, wobei sich die Mortalität mit steigender Punktzahl erhöht 3 . In der Tiermedizin werden die Kriterien oftmals aus der Humanmedizin übernommen, wobei der Versuch, neue klinische und biochemische Parameter mitaufzunehmen, bis dato keine signifikante Verbesserung in der praktischen Anwendung erbracht hat 2 4 .

Hinsichtlich der Therapie einer Sepsis gilt die Blutkultur immer noch als Goldstandard zur Identifizierung der beteiligten Erreger. Als Nachteil ist hierbei die mitunter mehrere Tage andauernde Zeitspanne bis zum positiven Ergebnis der Kultur ( *time to positivity* , TTP) zu nennen, die sich aus der Dauer der Einsendung zum Labor, der sich anschließenden Inkubation sowie der Identifikation des Erregers zusammensetzt. Hinzu kommt die darauffolgende Erstellung von Antibiogrammen, die einen weiteren Tag in Anspruch nimmt. Aufgrund des meist kritischen Zustands der Patienten werden Blutproben für eine Kultur oftmals unter laufender antibiotischer Therapie entnommen, was die Wahrscheinlichkeit eines negativen Kulturergebnisses erhöhen kann 1 . Deshalb sollte sich die initiale Therapie auf die wahrscheinlichste Ursache stützen, was die Identifikation von Eintrittspforten bakterieller Erreger, sogenannter septischer Foki, ebenso mit einschließt wie die Kenntnis über die am häufigsten vorkommenden Isolate. In der hier vorliegenden Studie wurden Ergebnisse von Blutkulturen von Pferden ausgewertet, die in den Jahren 2022 bis 2024 bei Laboklin GmbH und Co. KG in Bad Kissingen analysiert wurden, um neben einer potenziellen Aussagekraft der Methodik auch eine Übersicht der Isolate darzustellen.

## Material und Methoden

### Studienzeitraum und Probenumfang

Die Studie umfasst insgesamt 199 Proben von 180 Pferden, aufgeteilt in 76 Fohlen und 104 adulte Tiere, die in den Jahren 2022 bis 2024 aus tierärztlichen Praxen an ein veterinärmedizinisches Fachlabor (Laboklin GmbH und Co. KG, Bad Kissingen) zur Untersuchung einer Blutkultur übermittelt wurden. Drei Viertel (77,4%) wurden von tierärztlichen Praxen aus Deutschland eingeschickt, die restlichen 22,6% von Praxen aus dem europäischen Ausland. Das durchschnittliche Alter der Tiere bei Probennahme lag bei 6 Jahren, wobei das jüngste Tier am Tag seiner Geburt beprobt wurde, das älteste Tier war bei Probennahme 25 Jahre alt. Grundsätzlich wurde Blut von einem Entnahmezeitpunkt für eine Kultur eingeschickt, entweder als Flaschenpaar (bestehend aus aerober und anaerober Flasche) oder in einer Peds-Flasche. Es lagen keine Angaben über den Entnahmeort (periphere Vene, zentralvenöser Zugang) vor, ebenso wenig über die Abnahmesituation (Desinfektionsmaßnahmen, Lagerung der Proben). Lediglich 19 Patienten wurde zweimal Blut im Abstand von 20 Minuten für eine Kultur entnommen, ebenfalls als Flaschenpaar. Diese Proben wurden noch einmal separat ausgewertet, um eine Aussage über eine mögliche Sensitivitätssteigerung bei konsekutiven Blutentnahmen zu treffen. Nicht eingeschlossen wurden Blutkulturflaschen, die anderes Material als Blut enthielten (Synovia, Punktate) sowie Proben, die nicht in einer Blutkulturflasche, sondern in anderen Behältnissen (Spritze, Blutentnahmeröhrchen) versendet wurden. Auch Abstriche von Blutproben wurden nicht berücksichtigt. Die Patientendaten wurden dem der Probe beigefügten Untersuchungsauftrag entnommen, soweit angegeben. Zusätzliche Angaben wurden im Anschluss über einen separat an die Praxen verschickten Fragebogen erhoben.

### Bakteriologische Untersuchung

Je nach Hersteller der Blutkulturflasche variierte die Inkubationszeit und das Ableseintervall der bakteriologischen Kultur. Handelte es sich um BD Bactec Flaschen (Becton Dickinson GmbH, Heidelberg, Deutschland), erfolgte die Inkubation bei 35°C im dafür vorgesehenen Gerät (BD Bactec FX40, Becton Dickinson GmbH) für eine maximale Dauer von 5 Tagen nach Ankunft der Probe. Dieses Blutkultursystem detektiert bakterielles Wachstum im enthaltenen Flüssigmedium über ein Fluoreszenzsignal, ausgelöst durch das dabei entstandene CO _2_ . Die Flasche wurde bei Detektion bakteriellen Wachstums unmittelbar entnommen und jeweils ein Tropfen pro Agarplatte mittels Dreiösenausstrich ausplattiert. Handelte es sich um eine aerobe Flasche (BD Bactec Plus Aerobic/F-Kulturfläschchen), wurden als Nährböden Columbia Schafblutagar und Endo-Agar (Becton Dickinson GmbH) verwendet. Die Inkubation erfolgte unter aeroben Bedingungen für 24 Stunden. Anaerobe Flaschen (BD Bactec Plus Anaerobic/F-Kulturfläschchen) wurden auf Schaedler-Agar sowie Schaedler Kanamycin-Vancomycin Agar (Becton Dickinson GmbH) ausgestrichen und unter anaeroben Bedingungen für insgesamt 96 Stunden inkubiert. Eine Ausnahme bildeten Peds-Flaschen (BD Bactec Peds Plus/F-Kulturfläschchen), die sich für Patienten mit einem Körpergewicht von weniger als 20 kg eignen, da sie statt des üblichen Probenvolumens von 8–10 ml Blut mit einem geringeren Probenvolumen von 3 ml auskommen und in der Tiermedizin vor allem bei kleineren Hunderassen sowie Katzen eingesetzt werden. Bei diesen entfällt zudem die Notwendigkeit des ansonsten eingesetzten Flaschenpaars aus aerober und anaerober Kulturflasche, da das Probenmaterial sowohl aerob als auch anaerob kultiviert werden kann, wie in dieser Studie durchgeführt. Bei Blutkulturflaschen der Firma Thermo Fisher Scientific (Thermo Scientific Oxoid Signal, Fisher Scientific, Schwerte, Deutschland) ist ein Adapter aufgesetzt, in den die Probenflüssigkeit bei bakteriellem Wachstum aufsteigt, ausgelöst durch entstandenes CO _2_ . Sie wurden in der vorliegenden Studie direkt nach Probenankunft im Labor, unmittelbar nach positivem Wachstumssignal, oder – sollte dieses ausgeblieben sein – nach 7 Tagen noch einmal für die bakteriologische Kultur ausgestrichen. Nährböden und Inkubationszeiten entsprachen denen, die bei Verwendung von BD Bactec Flaschen eingesetzt wurden. Alle Isolate einer Probe wurden mithilfe eines Massenspektrometers (MALDI Biotyper sirius one, Bruker Corporation, Billerica, MA) differenziert und der Gehalt jedes Isolats semiquantitativ erfasst.

### Fragebogen

Da die in der Studie eingeschlossenen Proben als Routineproben ins Diagnostiklabor eingegangen waren, lag in den meisten Fällen keine Information bezüglich der klinischen Symptomatik des Patienten vor. Um einzuordnen, ob es sich um eine Sepsis handelte, wurde daher nachträglich ein Fragebogen an die jeweiligen Praxen verschickt, in denen mögliche Kriterien einer SIRS abgefragt wurden. Dazu zählten Fieber oder Hypothermie, Tachypnoe, Tachykardie, Leukozytose, Leukopenie oder Linksverschiebung. In Anlehnung an vorherige Studien wurde angenommen, dass beim Vorliegen von mindestens 2 positiven SIRS-Kriterien eine Sepsis vorlag 2 5 . Des Weiteren wurde abgefragt, ob der Patient zum Zeitpunkt der Entnahme mit Medikamenten vorbehandelt war, die das Ergebnis der Blutkultur beeinflussen könnten, und die Präparate in die Kategorien Antibiotika, Corticosteroide und Antiphlogistika unterteilt. Anhand der Frage, ob der Patient sich in stationärer Behandlung befand und die Infektion überlebt hat, sollten Rückschlüsse auf den Schweregrad der Infektion sowie die Mortalität gezogen werden.

### Statistische Auswertung

Die Daten wurden mithilfe von Microsoft Excel (Version 2502, Microsoft Office Home and Business 2021) aufgearbeitet und durch deskriptive Statistik ausgewertet.

## Ergebnisse

### Ergebnisse der bakteriologischen Untersuchung

**Abb. 1:**
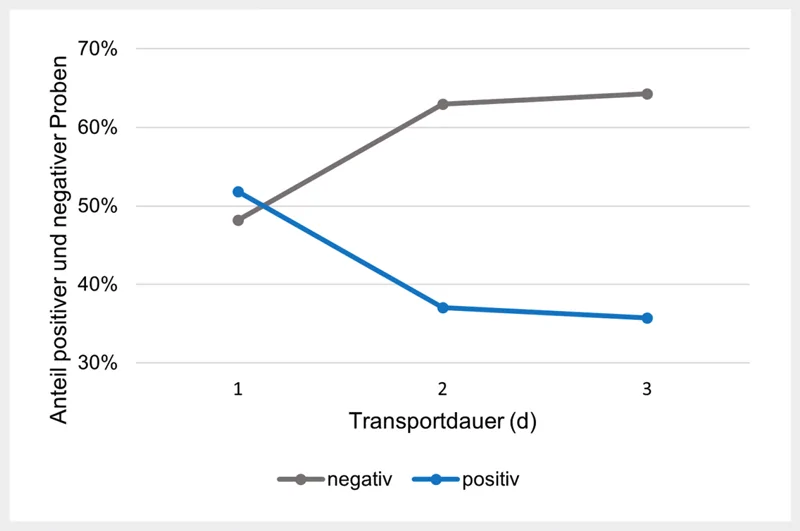
Anteil bakteriologisch positiver und negativer equiner Blutkulturen (%) in Abhängigkeit von der Transportdauer zum Labor (in Tage, n=151 Blutproben). Quelle: M. Geisler. **Fig. 1** Proportion of bacteriologically positive and negative equine blood culture samples (%) depending on the duration of transport to laboratory (in days, n=151 blood samples). Source: M. Geisler.

Die 180 Patienten setzten sich aus 76 Fohlen (42,2%) und 104 adulten Pferden (57,8%) zusammen. Die Mehrheit der Proben erreichte das Labor am Tag nach der Blutentnahme, woraus eine durchschnittliche Transportdauer von 1,4 Tagen resultierte. Die längste Transportdauer betrug 5 Tage. Die Zeitspanne zwischen Start der Inkubation im Labor und positivem Wachstumssignal ( *time to positivity* , TTP) betrug bei der aeroben Kultur durchschnittlich 2,9 Tage und bei der anaeroben Kultur 11,3 Tage. Bei den Proben, die am Tag nach der Blutentnahme im Labor eintrafen, war der Anteil an positiven und negativen Proben nahezu gleich (51,8% vs. 48,2%). Je länger die Transportdauer, desto höher fiel der Anteil negativer Proben aus und stieg auf 64,3% bei den Proben an, die nach 3 Tagen das Labor erreichten. Dagegen sank der Anteil an positiven Proben bis auf 35,7% ab ( Abb. 1 ).

**Abb. 2:**
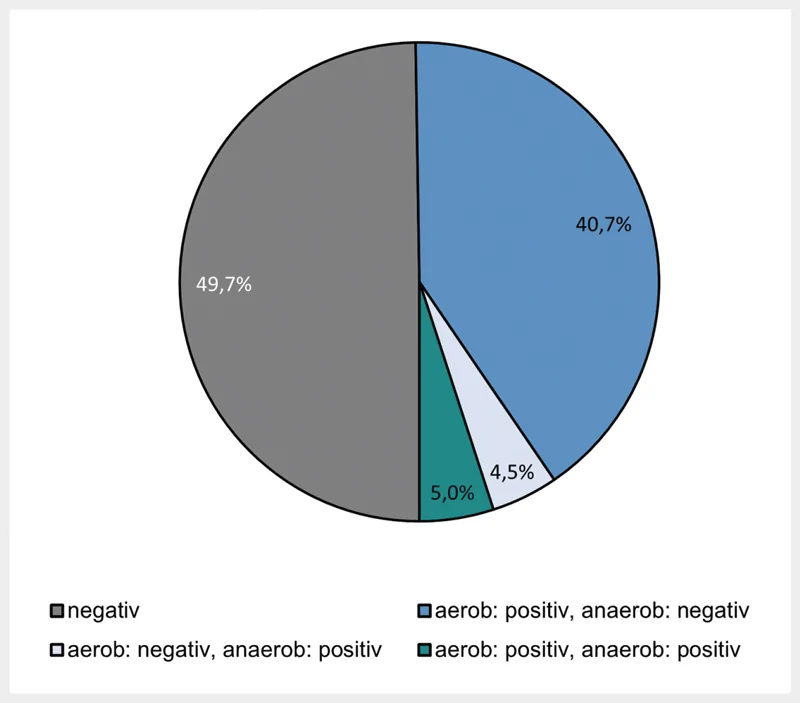
Anteil bakteriologisch positiver und negativer equiner Blutkulturproben (%) bezogen auf die Gesamtprobenzahl. Aufteilung der positiven Proben in aerobe und anaerobe Kultur (n=199 Blutproben). Quelle: M. Geisler. **Fig. 2** Proportion of bacteriologically positive and negative equine blood culture samples (%) based on total number of samples. Positives samples are divided in aerobic and anaerobic culture (n=199 blood samples). Source: M. Geisler.

**Abb. 3:**
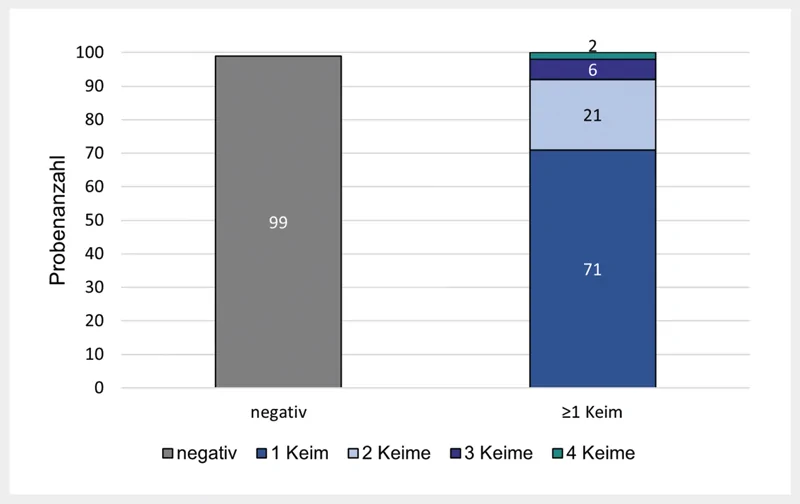
Anzahl bakteriologisch negativer und positiver equiner Blutkulturproben sowie Anzahl der davon mono- und polymikrobiellen Proben (n=199 Blutproben). Quelle: M. Geisler. **Fig. 3** Number of bacteriologically negative and positive equine blood culture samples, divided in mono- and polymicrobial samples (n=199 blood samples). Source: M. Geisler.

Wie in Abb. 2 dargestellt war bei der Hälfte der Proben (n=99, 49,7%) kein bakterielles Wachstum nachweisbar, weder in der aeroben noch in der anaeroben Kultur. Von den restlichen 100 Proben (50,3%) waren 81 positiv in der aeroben und in der anaeroben Kultur negativ. Demgegenüber waren 9 Proben negativ in der aeroben und positiv in der anaeroben Kultur. Lediglich 10 Proben wiesen sowohl in der aeroben als auch der anaeroben Kultur ein positives Ergebnis auf. Vergleicht man die Anzahl der in einer Probe nachgewiesenen bakteriellen Isolate, waren (unabhängig von aerober oder anaerober Kultur) von den 100 positiven Proben 71 monomikrobiell, die restlichen 29 Proben polymikrobiell. Diese 29 Proben gliederten sich weiter in 21 Proben, bei denen 2 Keime nachgewiesen wurden, 6 Proben mit 3 Keimen und 2 Proben mit 4 Keimen auf ( Abb. 3 ).

Tab. 1 zeigt die in der bakteriologischen Untersuchung nachgewiesenen Isolate, aufgeteilt nach aerober und anaerober Kultur. Insgesamt wurden mehr Gram-positive (56,1%) als Gram-negative Bakterien (35,8%) isoliert, sowohl bei der Gruppe der Fohlen (59,3% vs. 40,7%) als auch bei der Gruppe der adulten Pferde (62,5% vs. 37,5%). Am häufigsten wurden in der aeroben Kultur Staphylokokken (30,2%) isoliert, wobei Koagulase-negative Spezies gegenüber Koagulase-positiven Spezies dominierten. Dahinter folgten *Escherichia coli (E. coli)* und *Acinetobacter* spp. mit jeweils 9,4% sowie Streptokokken (7,5%). *Actinobacillus equuli* machte 4,7% der gefundenen Isolate aus und wurde sowohl bei Fohlen als auch bei Adulten nachgewiesen. *Rhodococcus equi* dagegen wurde nur dreimal ausschließlich bei Fohlen isoliert (2,8%). Strikte Anaerobier, die mit insgesamt 16% nur einen kleinen Teil der Isolate darstellten, wurden 17-mal nachgewiesen, wobei hauptsächlich *Cutibacterium* spp. (41,2%), *Bacteroides* spp. (17,6%) und *Fusobacterium* spp. (5,9%) identifiziert wurden.

**Table:** **Tab. 1** Übersicht der aus equinen Blutkulturproben isolierten Bakterien, unterteilt nach aerober und anaerober Kultur sowie nach Fohlen und adulten Pferden (n=123 Isolate). **Table 1** Overview of bacterial isolates found in equine blood cultures, divided in aerobic and anaerobic culture as well as foals and adult horses (n=123 isolates).

Isolate	Anzahl	(%)	Anzahl	(%)	Anzahl	(%)
Aerobe Kultur	Gesamt		Adult		Fohlen	
***Staphylococcus*** **spp.**	**32**	**30,2**	**13**	**26,5**	**19**	**33,3**
Koagulase-negative Staphylokokken	25	23,6	10	20,4	15	26,3
*S. aureus*	4	3,8	1	2,0	3	5,3
*S. haemolyticus*	2	1,9	1	2,0	1	1,8
*S. pseudintermedius*	1	0,9	1	2,0	0	0,0
**Enterobacterales**	**20**	**18,9**	**8**	**16,3**	**12**	**21,1**
*Escherichia coli*	10	9,4	4	8,2	6	10,5
*Pantoea* spp.	4	3,8	1	2,0	3	5,3
*Citrobacter* spp.	2	1,9	0	0,0	2	3,5
*Salmonella* spp.	1	0,9	1	2,0	0	0,0
*Klebsiella* spp.	1	0,9	1	2,0	0	0,0
*Raoultella* spp.	1	0,9	0	0,0	1	1,8
*Providencia* spp.	1	0,9	1	2,0	0	0,0
***Moraxellaceae***	**11**	**10,4**	**6**	**12,2**	**5**	**8,8**
*Acinetobacter* spp.	10	9,4	5	10,2	5	8,8
*Psychrobacter* spp.	1	0,9	1	2,0	0	0,0
***Streptococcus*** **spp.**	**8**	**7,5**	**6**	**12,2**	**2**	**3,5**
α-hämolysierende Streptokokken	5	4,7	3	6,1	2	3,5
β-hämolysierende Streptokokken	2	1,9	2	4,1	0	0,0
anhämolysierende Streptokokken	1	0,9	1	2,0	0	0,0
***Paeni-/Bacillaceae***	**7**	**6,6**	**4**	**8,2**	**3**	**5,3**
*Bacillus* spp.	4	3,8	1	2,0	3	5,3
*Paenibacillus* spp.	3	2,8	3	6,1	0	0,0
***Micrococcaceae***	**4**	**3,8**	**3**	**6,1**	**1**	**1,8**
*Micrococcus* spp.	2	1,9	2	4,1	0	0,0
*Pseudarthrobacter* spp.	2	1,9	1	2,0	1	1,8
***Pasteurellaceae***	**5**	**4,7**	**2**	**4,1**	**3**	**5,3**
*Actinobacillus* spp.	5	4,7	2	4,1	3	5,3
***Enterococcaceae***	**4**	**3,8**	**2**	**4,1**	**2**	**3,5**
*Enterococcus* spp.	4	3,8	2	4,1	2	3,5
***Aerococcaceae***	**4**	**3,8**	**2**	**4,1**	**2**	**3,5**
*Aerococcus* spp.	4	3,8	2	4,1	2	3,5
***Nocardiaceae***	**3**	**2,8**	**0**	**0,0**	**3**	**5,3**
*Rhodococcus* spp.	3	2,8	0	0,0	3	5,3
***Pseudomonadaceae***	**3**	**2,8**	**1**	**2,0**	**2**	**3,5**
*Pseudomonas* spp.	3	2,8	1	2,0	2	3,5
***Flavobacteriaceae***	**1**	**0,9**	**1**	**2,0**	**0**	**0,0**
*Capnocytophaga* spp.	1	0,9	1	2,0	0	0,0
Sonstige (nicht weiter differenziert)	4	3,8	1	2,0	3	5,3
**Gesamt**	**106**	**100**	**49**	**100**	**57**	**100**
**Anaerobe Kultur**	**Gesamt**		**Adult**		**Fohlen**	
*Cutibacterium* spp.	7	41,2	3	27,3	4	66,7
Strikte Anaerobier, nicht weiter differenziert	6	35,3	5	45,5	1	16,7
*Bacteroides* spp.	3	17,6	3	27,3	0	0,0
*Fusobacterium* spp.	1	5,9	0	0,0	1	16,7
**Gesamt**	**17**	**100**	**11**	**100**	**6**	**100**

**Abb. 4:**
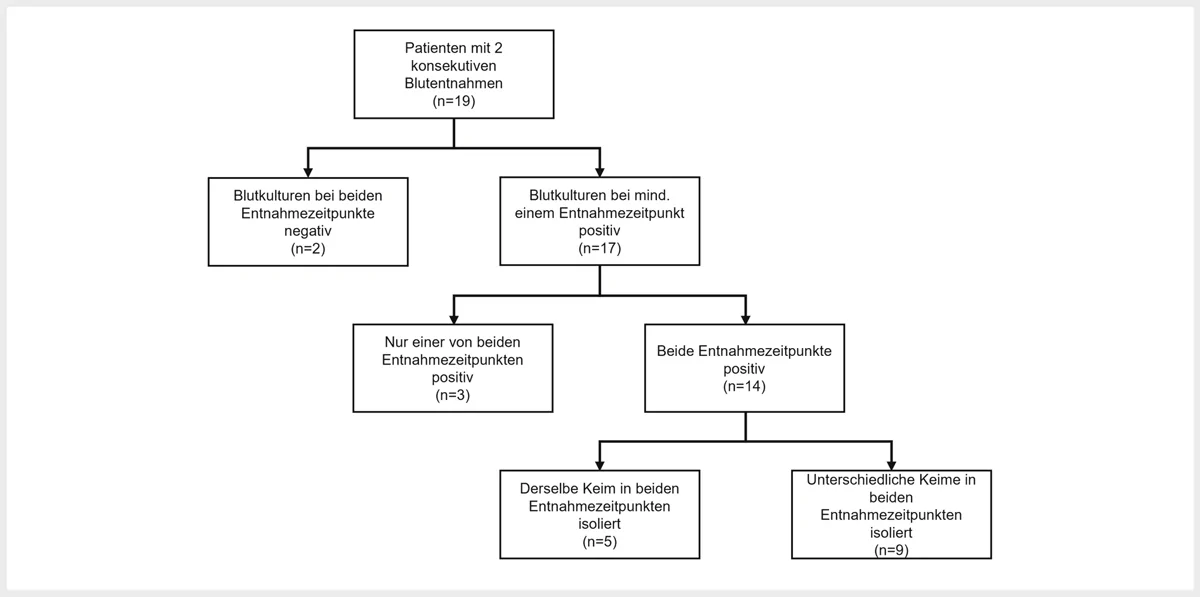
Flussdiagramm: Ergebnisse der bakteriologischen Kultur von Pferden mit 2 aufeinanderfolgenden Blutentnahmen. Gesonderte Betrachtung des jeweiligen Entnahmezeitpunkts in Bezug auf die isolierten Bakterien (n=19 Patienten). Quelle: M. Geisler. **Fig. 4** Flowchart: Bacteriologic results of equine patients with 2 consecutive blood culture samples. Separate analysis according to sampling time point in relation to bacterial isolates (n=19 patients). Source: M. Geisler.

Bei einem Teil der Patienten erfolgten 2 Blutentnahmen in einem Abstand von 20 Minuten. Diese Gruppe aus 19 Tieren (17 Fohlen, 2 Adulte) wurde im Hinblick auf die isolierten Erreger gesondert betrachtet. Wie in Abb. 4 dargestellt, wiesen 3 Patienten (18%) jeweils eine positive und eine negative Entnahme auf, wohingegen bei 14 Patienten (82%) beide Entnahmezeitpunkte positiv ausfielen. Hiervon wurden bei 4 Patienten nur Gram-positive (29%), bei 3 Patienten (21%) nur Gram-negative und bei 7 Patienten (50%) sowohl Gram-positive als auch -negative Keime nachgewiesen. In 5 Fällen (36%) konnte ein Keim zu beiden Entnahmezeitpunkten nachgewiesen werden, wobei die Blutkultur nur bei einem dieser Patienten monomikrobiell ausfiel ( **Zusatztab. 1** ).

### Auswertung des Fragebogens

Die 180 Patienten stammten aus 33 einsendenden tierärztlichen Praxen, die alle sowohl schriftlich als auch telefonisch kontaktiert wurden. Insgesamt wurden 46 Fragebögen aus 32 teilnehmenden Praxen zurückerhalten, auf die sich die im Folgenden dargestellten Daten beziehen.

**Abb. 5:**
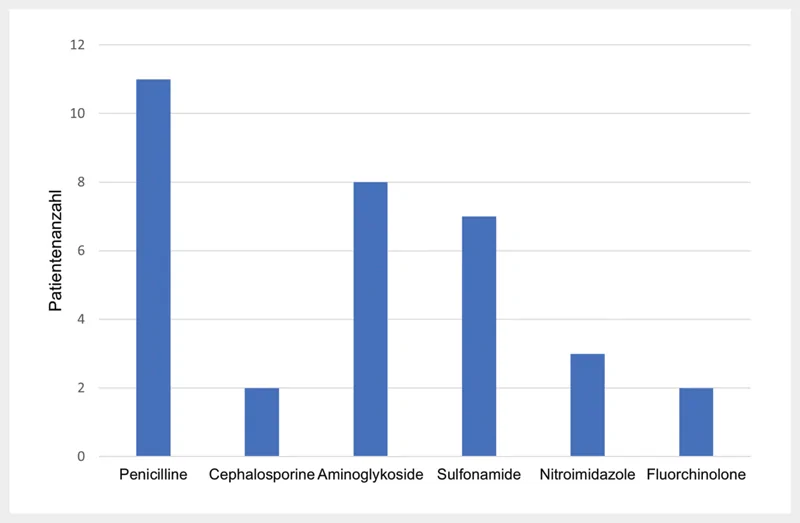
Ergebnisse der bakteriologischen Kultur von Blutkulturproben von Pferden in Abhängigkeit vom Vorliegen von SIRS-Kriterien und antibiotischer Vorbehandlung (n=46 Patienten). Quelle: M. Geisler. **Fig. 5** Bacteriologic culture results of equine blood culture samples in relation to SIRS-criteria and prior antibiotic treatment (n=46 patients). Source: M. Geisler.

**Abb. 6:**
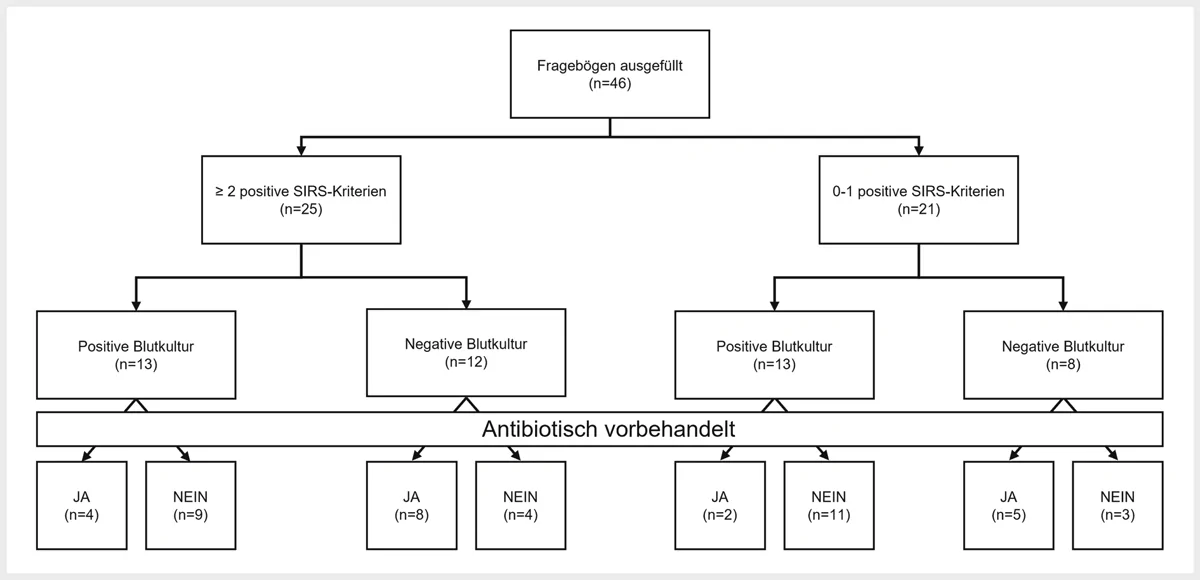
Übersicht der zum Zeitpunkt der Blutkulturentnahme eingesetzten Antibiotika bei Pferden, aufgeteilt in Wirkstoffgruppen (n=19 Patienten). Quelle: M. Geisler. **Fig. 6** Antibiotics administered at the time of blood culture collection in horses, grouped by antimicrobial class (n=19 patients). Source: M. Geisler.

Fast die Hälfte der Tiere (47,8%) war zum Zeitpunkt der Blutentnahme nicht vorbehandelt, demgegenüber hatten 41,3% Antibiotika, Corticosteroide (13,0%) oder nicht steroidale Antiphlogistika (28,3%) bzw. eine Kombination daraus erhalten. Bei denjenigen, die zum Zeitpunkt der Probenentnahme nicht antibiotisch vorbehandelt waren, fielen 74,1% der Proben positiv aus, bei den antibiotisch vorbehandelten Tieren waren es 31,6% der Proben ( Abb. 5 ). Bezogen auf Antibiotika wurden β-Lactame bei 13 Tieren (68,4%) und damit am häufigsten eingesetzt, gefolgt von Aminoglykosiden (8 Tiere; 42,1%) und Sulfonamiden (7 Tiere; 36,8%) ( Abb. 6 ). Acht (42,1%) der antibiotisch vorbehandelten Tiere erhielten einen Wirkstoff, 7 Tiere (36,8%) 2 verschiedene Wirkstoffe und 4 Tiere (21,1%) mehr als 2 verschiedene Wirkstoffe. Die am häufigsten eingesetzten Kombinationen aus 2 Wirkstoffen waren Aminoglykoside + Sulfonamide, β-Lactame + Sulfonamide sowie β-Lactame + Aminoglykoside (je dreimal eingesetzt).

Insgesamt zeigten 25 Tiere (54,3%) mindestens 2 positive SIRS-Kriterien. Von diesen befanden sich 20 (80,0%) in stationärer Behandlung, 6 Tiere (24,0%) verstarben. Bezogen auf die Gesamtzahl an ausgewerteten Fragebögen befanden sich 38 Tiere (82,6%) in stationärer Behandlung, wobei 7 Tiere (18,4%) verstarben oder euthanasiert werden mussten.

**Abb. 7:**
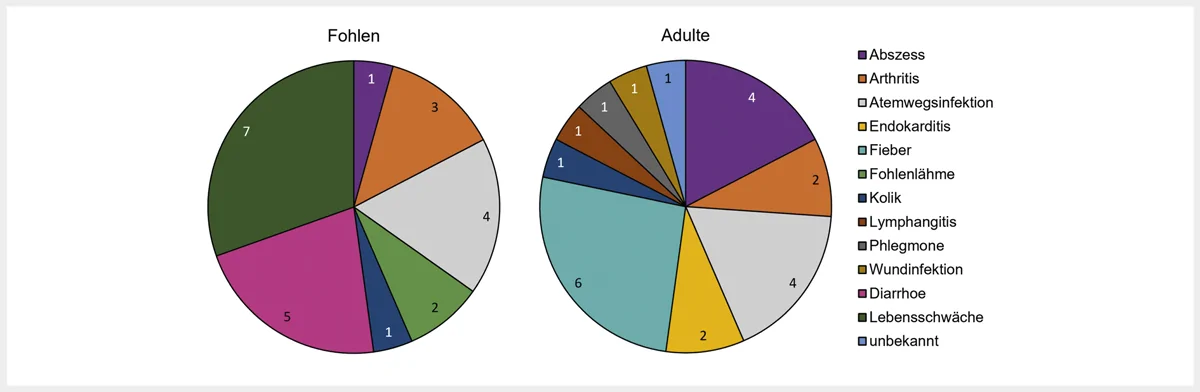
Gründe für die Untersuchung einer Blutkultur bei Pferden, getrennt dargestellt für die Anzahl der Fohlen und adulten Tiere (n=46 Patienten). Quelle: M. Geisler. **Fig. 7** Reasons for initiating the sampling of a blood culture in horses, presented separately for foals and adult horses (n=46 patients). Source: M. Geisler.

Bei adulten Pferden wurde als Grund für die Blutentnahme am häufigsten Fieber unbekannter Genese, teilweise rezidivierend, angegeben (27,3%). Es folgten Atemwegsinfektionen und Abszesse mit je 18,2%. Bei Fohlen wurde Lebensschwäche (29,2%), gefolgt von Atemwegsinfektionen und Arthritiden (16,7% und 12,5%) am häufigsten genannt. Im direkten Vergleich waren bei den adulten Tieren mehr unterschiedliche Gründe angegeben als bei Fohlen ( Abb. 7 ).

## Diskussion

Eine bakterielle Sepsis gilt als eine der Hauptursachen für eine erhöhte Sterblichkeit beim neonatalen Fohlen. Haupteintrittspforten für Erreger sind neben Nabel oder Plazenta auch die Schleimhäute des Gastrointestinal- und Respirationstrakts. Eine unzureichende Desinfektion des Nabels und mangelnde Sauberkeit der Umgebung gelten als Risikofaktoren. Hinzu kommt, dass die als Darmbarriere bezeichnete Schutzschicht während der ersten 24 Lebensstunden noch durchlässig ist und Bakterien somit leicht in die Zirkulation übertreten können. Neben einer Bakteriämie führt dies im Fall von Gram-negativen Erregern oftmals zu einer Endotoxämie durch das in der Zellwand enthaltene Lipopolysaccharid (LPS) 6 . Beim adulten Pferd werden als Gründe für eine Bakteriämie unter anderem Colitis, zugrundeliegende Kolik oder medizinische Eingriffe wie Zahnextraktionen genannt 8 , was in dieser Auswertung zumindest teilweise bestätigt werden konnte. Die Mortalitätsrate durch Septikämien bei neonatalen Fohlen variiert je nach Studie zwischen 30–85%, bei adulten Tieren ist sie variabler und von der Art und dem Schweregrad der Infektion abhängig 2 . In der vorliegenden Studie verstarben 8 von 46 Tieren (17,4%), was angesichts der Tatsache, dass 25 Tiere mind. 2 positive SIRS-Kriterien zeigten und sich 38 Tiere in stationärem Aufenthalt befanden, vergleichsweise niedrig erscheint. Es konnte kein Zusammenhang hinsichtlich einer erhöhten Sterblichkeit und der Anzahl positiver SIRS-Kriterien ausgemacht werden, was darauf zurückgeführt werden kann, dass diese aus der Humanmedizin abgeleitet werden und eine mitunter geringe Sensitivität aufweisen 1 2 .

Ein negativer Aspekt der bakteriellen Kultur ist die bestehende Diskrepanz zwischen einem negativen Ergebnis trotz vorliegendem Sepsisverdacht. Als Gründe werden neben der mitunter langen Transportdauer der Proben eine zu geringe Anzahl an im Blut zirkulierender Erreger diskutiert. Die Bakteriämie tritt intermittierend auf, was sich in den hier vorliegenden Ergebnissen widerspiegelt. So zeigten von den 19 Patienten, bei denen Kulturergebnisse von 2 Entnahmezeitpunkten vorlagen, lediglich 2 Patienten (11%) ein vollständig negatives Ergebnis. Bei 3 Patienten (18%) fiel einer der beiden Entnahmezeitpunkte in der Kultur positiv, der andere negativ aus. Es ist daher anzunehmen, dass im Fall von nur einer Blutentnahme der Anteil der Patienten mit negativem Kulturergebnis höher ausgefallen wäre, was die Bedeutung von konsekutiven Blutentnahmen in kurzem zeitlichen Abstand nochmals hervorhebt 1 . Ein humanmedizinischer Assay von De Plato et al. 8 betont gar die Relevanz einer dritten Blutentnahme desselben Patienten, ohne die laut Meinung der Autoren in 7,9% der Fälle die Kultur ein falsch negatives Ergebnis zur Folge gehabt hätte.

Der Anteil positiver Proben fiel in der vorliegenden Studie mit 50,3% deutlich höher als in früheren Studien aus, deren Positivitätsraten zwischen 25 und 44% schwankten 7 . Dies kann zum einen auf die kurze Transportdauer der Proben zurückzuführen sein: Die Mehrheit der Proben (55,3%) erreichte das Labor bereits am Tag nach der Probennahme, was mit einem höheren Anteil positiver Proben korrelierte. Mit zunehmender Transportdauer stieg der Anteil negativer Proben an, was die Relevanz eines unverzüglichen Transports der Proben an das untersuchende Labor noch einmal hervorhebt 8 . Zum anderen nimmt die Art der Probennahme Einfluss auf die Ergebnisse der bakteriologischen Kultur. Hier ist als wesentliche Limitation der Studie zu nennen, dass keinerlei Daten hinsichtlich der Aseptik während der Probennahme oder der Entnahmestelle selbst vorlagen. Auch das Probenvolumen in der Kulturflasche spielt eine Rolle. Während das Überfüllen der Flasche zu einem falsch positiven Wachstumssignal im Inkubator ohne tatsächlich vorliegendes bakterielles Wachstum führen kann, verlängert ein zu geringes Probenvolumen das Zeitintervall bis zum Wachstumssignal ( *time to positivity* , TTP) oder zieht gar falsch negative Ergebnisse nach sich 8 . In der Veterinärmedizin existieren bezüglich des Probenvolumens keine standardisierten Empfehlungen. Ein Vergleich vorangegangener Studien zeigte, dass bei Fohlen oftmals zwischen 5–20 ml einmalig entnommen werden. Da Fohlen im Vergleich zu Neonaten anderer Spezies ein höheres Körpergewicht aufweisen, wird ein Probenvolumen von 30–60 ml grundsätzlich nicht als problematisch angesehen [7]. In einer humanmedizinischen Studie konnte gezeigt werden, dass eine Erhöhung des Probenvolumens von 10 auf 30 ml die Positivitätsrate um 61% steigerte. Insgesamt beschrieben die Autoren eine Steigerung der Sensitivität von 3% pro Milliliter mehr abgenommenem Blut 9 . Ob das Probenvolumen in den hier untersuchten Proben eingehalten wurde, was die Ergebnisse hätte beeinflussen können, konnte nachträglich nicht überprüft werden und stellt eine weitere Limitation der Studie dar.

Die in Tab. 1 dargestellten bakteriellen Isolate spiegeln teilweise die Ergebnisse vorheriger Studien zur Auswertung von Blutkulturen wider. So werden bei Fohlen als Gram-positive Erreger vor allem Staphylokokken, Streptokokken und Enterokokken genannt 2 10 , die auch in der vorliegenden Studie isoliert wurden. Vergleicht man heutige Ergebnisse mit denen älterer Studien, so zeigt sich zudem ein Anstieg der Gram-positiven Isolate. Als Grund kann eine steigende Resistenzproblematik, vor allem bei Enterokokken, diskutiert werden. Diese machten in dieser Auswertung mit 3,8% nur einen kleinen Teil der Isolate aus, allerdings ist der Studienzeitraum zu klein um einen Trend auszumachen und Resistenzen wurden nicht ausgewertet. Dass insgesamt mehr Gram-positive als Gram-negative Erreger isoliert wurden, steht einer Studie von Theelen et al. 10 gegenüber, in der der Anteil Gram-negativer Isolate mit 70% deutlich höher war als der der Gram-positiven (30%). Diese Diskrepanz könnte darauf zurückzuführen sein, dass in der dortigen Studie nur Fohlen inkludiert wurden, die klinische Anzeichen einer Sepsis zeigten, wohingegen in der vorliegenden Studie alle Isolate abgebildet sind, unabhängig davon, ob klinisch gesehen eine Sepsis vorlag oder nicht. Als bedeutsame Gram-negative Erreger werden neben *E. coli* beim septischen Fohlen vor allem *Actinobacillus* , *Klebsiella, Salmonella, Enterobacter* und *Proteus* genannt. Diese Spezies konnten hier bis auf *Actinobacillus* spp., der bei 4,2% der Fohlen und 1,8% der adulten Pferde nachgewiesen wurde, nicht bestätigt werden. Dennoch stellte die Ordnung der Enterobacterales hinsichtlich der Anzahl der Isolate und Zahl der betroffenen Patienten jeweils die zweitgrößte Gruppe nach den Staphylokokken dar. Bezogen auf die anaerobe Kultur ist der Anteil der negativen Proben in der Studie von Theelen et al. 10 deutlich höher als in der aeroben Kultur, was den Ergebnissen dieser Studie entspricht. Giancola et al. 7 stellen daher die Relevanz der anaeroben Kultur infrage und schlagen aufgrund des limitierten Blutentnahmevolumens vor, diese zugunsten von mehr aeroben Flaschen entfallen zu lassen.

Eine Einordnung der Isolate in „klinisch relevant“ und „Kontamination“ wird in dieser Studie bewusst nicht vorgenommen. Spezies wie Koagulase-negative Staphylokokken, *Bacillus* spp., *Corynebacterium* spp. sowie *Propionibacterium* spp. werden zwar generell als häufige Kontaminanten in Blutkulturen angesehen, besonders wenn diese nur in einer von mehreren Entnahmezeitpunkten /-stellen auftreten, allerdings spricht das Auftreten derselben Spezies in konsekutiven Proben für eine vorliegende Bakteriämie durch diesen Erreger. Theoretisch kann somit jedes Isolat als Kontamination gezählt werden – unabhängig von der zugrundeliegenden Kategorisierung der aktuellen Literatur – sollte es nur in einer Flasche gefunden worden sein 5 . Da hier bis auf 19 Tiere mit mehreren Entnahmezeitpunkten sonst nur einmalig Blut für eine Kultur entnommen wurde, entfällt eine dahingehende Bewertung.

Eine initiale Antibiotikatherapie ist aufgrund des mitunter lebensbedrohlichen Zustands des Patienten meist unerlässlich und deren Einleitung somit bereits vor Erhalt der Kulturergebnisse erforderlich. Laut Literatur wird bei septischen Fohlen eine Kombination aus Aminoglykosid und β-Lactamen oder Cephalosporinen der 3. Generation empfohlen. Dies zeigte auch die Auswertung der hier vorliegenden Daten, in denen diese Wirkstoffgruppen (entweder einzeln oder in Kombination) am häufigsten verabreicht wurden. Dass sich eine Probennahme unter laufender Antibiose negativ auf das bakteriologische Ergebnis auswirken kann 1 , konnte ebenfalls bestätigt werden (31,6% positive Proben bei vorbehandelten Tieren versus 74,1% positive Proben bei nicht vorbehandelten Tieren).

## Notes

Fazit für die Praxis
Die Diagnose einer Sepsis basiert auf der klinischen Symptomatik, es gibt derzeit keinen speziellen Sepsistest, auch wenn die Serumkonzentrationen von Procalcitonin oder C-reaktivem Protein (CRP) hinweisend sein können 11 12 . Das Wissen um mögliche zugrundeliegende Erreger ist essenziell, um vor Vorliegen der Blutkulturergebnisse eine gezielte antibiotische Therapie einzuleiten. Antibiogramme helfen bei der Auswahl des geeigneten Antibiotikums in zweierlei Hinsicht: wenn die initiale Antibiose nicht anschlägt und wenn ein Wechsel zugunsten eines Antibiotikums mit engerem Wirkspektrum durchgeführt werden kann. Dies sollte wenn möglich auch immer erfolgen, um die Ausbildung weiterer Resistenzen zu verhindern. Eine transiente Bakteriämie von einer therapiebedürftigen Sepsis anhand eines positiven Blutkulturergebnisses unterscheiden zu können, ist oft schwierig. Daher sollten eine korrekte Entnahmetechnik (ausreichendes Probenvolumen, Aseptik bei Probennahme, korrekte Lagerung der Proben) angewandt werden und zusätzliche Untersuchungsergebnisse (biochemische Marker, Bildgebung, Zytologie) bei der Interpretation berücksichtigt werden. Alternativen zur kulturellen Untersuchung wie *multiplex polymerase chain reaction* (Polymerase-Kettenreaktion, PCR) oder Massenspektrometrie-gestützte Verfahren zur Erregeridentifikation sollten weiter ausgebaut werden. Diese setzen spezielles Equipment voraus und sind derzeit teils kostenintensiv, weshalb sie momentan noch nicht dem Standardprozedere in der Veterinärmedizin entsprechen 7 . Es kann aber davon ausgegangen werden, dass sie bei Etablierung deutlich verkürzte Untersuchungszeiten zum Wohl der Patienten ermöglichen werden.

## Zusätzliches Material

Zusätzliches Material Zusätzliches Material
