# Supplementary material for: Retrospective evaluation of equine blood cultures sampled between 2022 and 2024
Source: Tierarztl Prax Ausg G Grosstiere Nutztiere. 2026 May 7;54(4):253–61. [Article in German] doi: 10.1055/a-2848-1852 (PMC13493176; doi:10.1055/a-2848-1852)
Supplement: Supplementary file 1 [file 10-1055-a-2848-1852-28481852.pdf]

Zusatzmaterial

Zusatztab. 2 Ergebnisse der bakteriologischen Kultur von Pferden mit 2 konsekutiven Blutentnahmen (n=19 Patienten). Quelle: M. Geisler.

Supplementary Table 2 Results of bacteriological culture from horses with 2 consecutive blood sampling time points (n= 19 patients). Source: M. Geisler.

Patient	Entnahme-zeitpunkt	Aerobe Kultur			Anaerobe Kultur
1	1	***Staphylococcus xylosus***	***Staphylococcus hämolyticus***	negativ	negativ
	2	***Staphylococcus xylosus***	negativ	negativ	negativ
2	1	***Aerococcus urinaeequi***	***Acinetobacter* spp.**	negativ	negativ
	2	***Staphylococcus xylosus***	negativ	negativ	negativ
3	1	***Raoultella terrigena***	negativ	negativ	negativ
	2	***Staphylococcus sciuri***	negativ	negativ	**strikte Anaerobier**
4	1	***Escherichia coli***	***Acinetobacter lwoffii***	***Staphylococcus vitulinus***	negativ
	2	***Staphylococcus equorum***	negativ	negativ	negativ
5	1	***Acinetobacter lwoffii***	negativ	negativ	negativ
	2	***Bacillus thuringiensis***	negativ	negativ	negativ
6	1	***Paenibacillus* spp.**	negativ	negativ	negativ

	2	***Paenibacillus spp.***	***Streptococcus salivarius***	***Streptococcus equinus***	negativ
7	1	negativ	negativ	negativ	negativ
	2	negativ	negativ	negativ	negativ
8	1	***Staphylococcus xylosus***	negativ	negativ	negativ
	2	negativ	negativ	negativ	negativ
9	1	***Bacillus cereus***	negativ	negativ	negativ
	2	***Staphylococcus aureus (Methicillin resistent)***	***Enterococcus faecium***	negativ	negativ
10	1	***Staphylococcus hämolyticus***	***Pantoea agglomerans***	negativ	***Cutibacterium acnes***
	2	negativ	negativ	negativ	***Cutibacterium flaccumfaciens***
11	1	negativ	negativ	negativ	negativ
	2	***Citrobacter murliniae***	negativ	negativ	negativ
12	1	***Escherichia coli***	negativ	negativ	negativ
	2	***Acinetobacter pseudolwoffii***	negativ	negativ	negativ
13	1	***Citrobacter murliniae***	negativ	negativ	negativ
	2	***Citrobacter murliniae***	***Enterococcus mundtii***	negativ	***Cutibacterium acnes***
14	1	***Escherichia coli***	negativ	negativ	negativ
	2	***Escherichia coli***	negativ	negativ	negativ

15	1	***Staphylococcus* spp.**	**α-hämolysierende Streptokokken**	negativ	negativ
	2	***Staphylococcus sciuri***	***Staphylococcus xylosus***	negativ	negativ
16	1	***Pseudomonas fluorescens***	negativ	negativ	negativ
	2	***Staphylococcus equorum***	negativ	negativ	negativ
17	1	***Staphylococcus equorum***	negativ	negativ	negativ
	2	negativ	negativ	negativ	negativ
18	1	***Acinetobacter pseudolwoffii***	***Pseudomonas putida***	negativ	negativ
	2	***Acinetobacter pseudolwoffii***	negativ	negativ	negativ
19	1	negativ	negativ	negativ	negativ
	2	negativ	negativ	negativ	negativ
